# Catecholaminergic neurons orchestrate fasting-induced immune harmony

**DOI:** 10.52601/bpr.2024.240901

**Published:** 2024-02-29

**Authors:** Mengdi Guo, Weiyan Wang, Xiao Tu, Meiling Jiang, Cun-Jin Zhang

**Affiliations:** 1 Department of Neurology, Sichuan Provincial People's Hospital, University of Electronic Science and Technology of China, Chengdu 611731, China; 2 Department of Science and Technology, Sichuan Provincial People's Hospital, University of Electronic Science and Technology of China, Chengdu 611731, China

Dietary fasting is a well-established intervention for immunosuppressive therapy, but the underlying mechanism remains elusive. Host fitness to nutritional stress has been confirmed to initiate a cascade of adaptive responses mediated by the central nervous system, shedding new light on a neural link between fasting and immunity (de Cabo and Mattson 2019). In a recent study published in *Nature Neuroscience,* Wang *et al*. reveal a complex interplay among fasting, neuromodulation, and the immune system. While fasting remobilized the circulating T cell pool in a CXCR4/CXCL12 axis-dependent manner, continuous activation of ventrolateral medullary catecholaminergic neurons (CA^VLM^) mimicked the bone marrow homing effect and proved obvious relief in autoimmune diseases (Wang *et al.* 2024).

Compared with other immune organs, bone marrow is a nutrient-rich shelter for the mature immune cells faced with nutrient scarcity. Fasting is acknowledged to trigger these high-energy consumed cells colonized in bone marrow, a fundamental adaptation strategy for promoting extended lifespan and protective functions, raising interesting questions about the underlying regulatory mechanisms and consequences of inflammatory disease outcomes (Nagai *et al.* 2019). In the groundbreaking investigation, the authors demonstrated that this phenomenon largely relied on an undiscovered neural mechanism, which emphasized the central role of CA^VLM^ neurons in orchestrating immune responses during energy scarcity. Activated by 24-hour fasting, these neurons drove T cell homing to the bone marrow but decreasing in the blood, spleen, and inguinal lymph nodes. Selective ablation of CA^VLM^ neurons effectively demonstrated their sufficiency and necessity for fasting-induced T cell redistribution (Wang *et al.* 2024). Strikingly mirroring fasting effects, sustained activation of CA^VLM^ neurons with chemogenetics curtails T cell activation, proliferation, differentiation, and cytokine production in mouse models of pathogenic T cells mediated autoimmune diseases. Excessive immune responses were also consequently reduced in experimental autoimmune encephalomyelitis (EAE), psoriasis-like skin inflammation, and mBSA-induced delayed-type hypersensitivity (DTH), especially the percentages and numbers of pathogenic Th1 and Th17 cells (Wang *et al.* 2024).

In addition, the observed bone marrow homing effect was found to be controlled in a CXCR4/CXCL12 axis-dependent manner, which was potentially mediated by a CA^VLM^→CRH^PVN^ neural circuit governing adrenal corticosterone secretion through the hypothalamic‒pituitary‒adrenal axis (HPA) (Wang *et al.* 2024). CA^VLM^ neurons had an anatomical connection with corticotropin-releasing hormone neurons in the hypothalamic paraventricular nucleus (CRH^PVN^), a brain region implicated in immune regulation. Consistent with the projections from CA^VLM^ neurons to CRH^PVN^ neurons, and the known role of glucocorticoids in circulating T cell redistribution, stimulation of CA^VLM^ neurons or selective activation of the CA^VLM^→PVN neural circuit promoted corticosterone secretion from the adrenal glands and upregulated CXCR4 expression of T cells, triggering significant blood-to-bone marrow homing (Fig.1) (Wang *et al.* 2024). This collective evidence underscores the pivotal role of neuronal control in fasting-mediated immunosuppression, opening new avenues for understanding and potentially modulating immune responses under nutritional stress.

**Figure 1:**
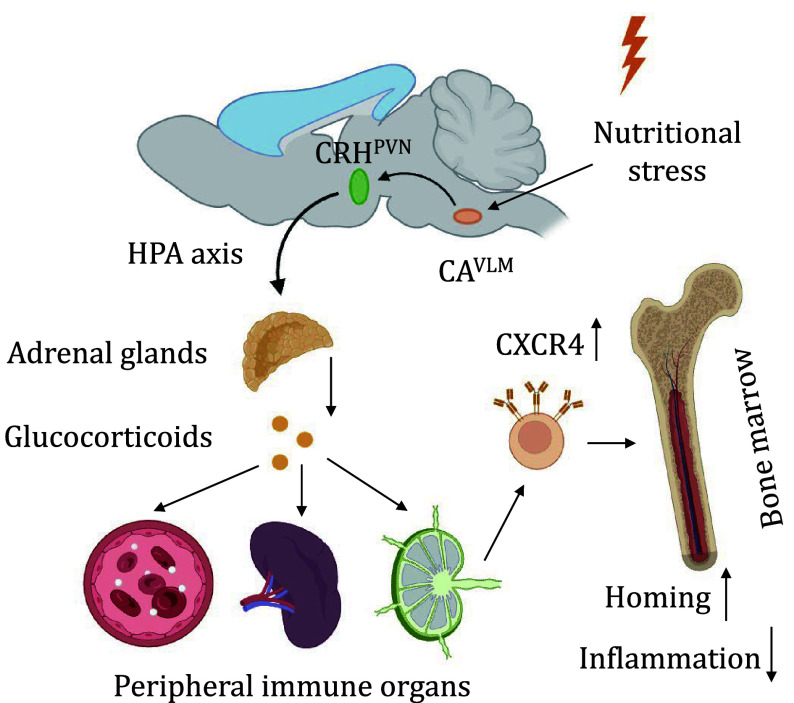
Neuronal regulation of the immune system during dietary fasting. Orexigenic CA^VLM^ neurons are activated during fasting and stimulate the release of glucocorticoids via the CA^VLM^→CRH^PVN^ neural circuit targeting the HPA axis, thereby driving T-cell homing to the bone marrow in a CXCR4/CXCL12 dependent manner and suppressing excessive inflammation

This research may hold considerable importance for a broad audience, encompassing individuals engaging in fasting for health advantages, and researchers specializing in neuroscience and immunity. In fact, the nervous system has long been implicated in orchestrating large-scale immune cell migration (Pavlov and Tracey 2017). Pioneering studies have revealed the intricate connection between the nervous and immune systems, highlighting how specific neural circuits and brain regions influence the stressed-induced, counter-directional shifts in leukocyte populations. A fasting/refeeding-induced mechanism underscores the temporal dynamics of diet in modulating monocyte lifespan, which also involves a CXCR4 response mediated by corticosteroid and monocytic glucocorticoid receptor NR3C1 via the HPA axis (Janssen *et al.* 2023). Poller *et al*. demonstrate that motor circuits induce rapid mobilization of neutrophils from the bone marrow to peripheral tissues, while the paraventricular hypothalamus controls monocyte and lymphocyte dynamics between secondary lymphoid organs, blood, and bone marrow through glucocorticoid signaling, finally participating in the altered susceptibility to infection, injury, and systematic inflammation (Poller *et al.* 2022). More evidence from diabetes research suggests a role for sympathetic neuronal activation in the generation of inflammatory myeloid cells from hematopoietic progenitor cells. Myeloid cell numbers strongly correlated with plasma norepinephrine levels in diabetic patients, and mice lacking tyrosine hydroxylase (TH)-producing leukocytes exhibited diminished granulocyte macrophage progenitor (GMP) proliferation, highlighting the role of catecholamines in modulating hematopoiesis and leukocyte hemostasis (Vasamsetti *et al.* 2018). In addition to the cooperation of neural catecholaminergic circuits, vagus nerve–splenic nerve reflex, Pavlovian conditioning and reward system, as well as brain integration of neuroimmune pathways are also involved in the neural regulation of immunity and inflammation (Carnevale *et al.* 2016; Schedlowski and Pacheco-López 2010; Udit *et al.* 2022). Understanding the depth and selectivity of the nervous system's regulatory control over immune function will be crucial for monitoring and modulating immune homeostasis and maximizing efforts in implementing effective strategies for aberrant immune responses.

To summarize, this research lays the foundation for future investigations into the involvement of diverse neuronal populations in regulating immune responses during fasting, and hints at the broader implications of orexigenic CA^VLM^ neurons in immune modulation, particularly in the context of various stress stimuli beyond fasting. Although the research has limitations in directly translating to human physiology, it offers valuable insights into the potential mechanisms underlying fasting-induced immune harmony and neural regulation on T cell function and distribution. Additional studies are required to investigate the implications for human health, dietary recommendations, and potential therapeutic interventions for inflammatory and autoimmune illnesses.

## Conflict of interest

Mengdi Guo, Weiyan Wang, Xiao Tu, Meiling Jiang and Cun-Jin Zhang declare that they have no conflict of interest.
